# Reversible Pulmonary Hypertension Associated with Whipple's Disease

**DOI:** 10.1155/2012/382460

**Published:** 2012-10-02

**Authors:** A. Villa, G. Nucera, A. Kostihova, A. Mazzola, P. Marino

**Affiliations:** ^1^U.O. Medicina d'Urgenza e Pronto Soccorso, A. O. Fatebenefratelli, 20123 Milano, Italy; ^2^U.O. Cardiologia, A. O. Fatebenefratelli, 20123 Milano, Italy

## Abstract

We describe a case of Whipple's disease with pulmonary hypertension in a 72-year-old woman in whom the pulmonary hypertension resolved completely after antibiotic therapy. She was admitted to study with a 2-months history of weight loss, diarrhoea, abdominal pain, asthenia, inappetence, and fever. She did not have dyspnoea or respiratory symptoms. A casual echocardiogram showed a pulmonary artery systolic pressure of 95 mmHg. Forty days after starting antibiotic therapy, an echocardiogram showed a complete normalisation of right ventricular involvement. Whipple's disease is a rare and multisystemic disorder in which pulmonary involvement is not a well-known finding. Although Whipple's disease is not generally considered as a possible cause of pulmonary hypertension, such awareness is important because it may be potentially resolved with antibiotic therapy.

## 1. Introduction

Whipple's disease is a rare and multisystemic disorder of infectious aetiology caused by *Tropheryma whipplei* which was originally described by Whipple [1].

Although the main manifestation of this disease is gastrointestinal, pulmonary involvement is a frequent but not well-known finding.

A chronic nonproductive cough, dyspnoea, and pleural chest pain have been reported more often than pleural effusion and nodular shadowing [2–4].

Pulmonary hypertension associated with Whipple's disease has been reported previously in rare cases [5–10].

We describe a case of Whipple's disease with pulmonary hypertension in a 72-year-old woman in whom the pulmonary hypertension resolved completely after antibiotic therapy.

## 2. Case Report

A 72-year-old woman was admitted to study with a 2-month history of weight loss, diarrhoea, abdominal pain, asthenia, inappetence, and fever. She was treated for depressive syndrome. She did not have dyspnoea or respiratory symptoms. 

Physical examination showed a pale and cachectic woman; cardiovascular, respiratory, abdominal, and neurological examination was normal; pretibial oedema was not present. 

The laboratory haematological values revealed microcytic hypochromic anaemia, and blood chemical values indicated malabsorption (potassium 2.96 mmol/L, protein 4.8 mg/dL, albumin 2.78 mg/dL, cholesterol 78 mg/dL, ferrum 18 mcg/dL, and ferritin 33 ng/mL).

Gastroduodenoscopic examination showed a spontaneous bleeding of gastric and duodenal mucosa. Histological tissue specimen showed periodic acid Schiff positive macrophages infiltration in the lamina propria. The microscopic observation demonstrated intracellular bacilli, as typical appearance of Whipple's disease. 

For an aspecific transient chest pain, an echocardiogram was performed, and it showed a mild right ventricular dilatation, a mild-severe tricuspid insufficiency with an estimated pulmonary artery systolic pressure of 95 mmHg.

Blood gas analysis revealed   PaO_2_ 76.7 mmHg and PaCO_2_ 20.5 mmHg. 

A chest angio-CT scan excluded pulmonary embolism. 

Tests for antinuclear antibodies, antineutrophil cytoplasmic antibodies, rheumatoid factor, HIV, and other viral diseases were negative.

After the diagnosis of Whipple's disease had been established, antibiotic therapy was started with ceftriaxone intravenously for 14 days followed by trimethoprim and sulphamethoxazole orally twice a day.

Few hours after the first dose of antibiotic treatment, the patient felt unwell and developed a fever of 39°C. The symptoms resolved after a short time, as described in the Jarisch-Herxheimer reaction. 

After the beginning of antibiotic treatment, the diarrhoea resolved completely and the general conditions of the patient rapidly improved. 

After forty days, the echocardiogram showed a complete normalisation of right ventricular involvement and the pulmonary artery systolic pressure was 29 mmHg.

Hemodynamic assessment was not performed for a rapid improvement of clinical conditions with a complete normalisation of echocardiographic parameters.

After 8 months of therapy, the patient was asymptomatic with normal weight and healthy appetite.

## 3. Discussion

Although the involvement of the lung as a site of this disease was reported in Whipple's first description in 1907 [1], Whipple's disease is not generally considered as a possible cause of pulmonary hypertension. To the best of our knowledge, the finding of the Whipple-associated pulmonary hypertension has been reported in only 6 cases in the literature [5–10]. In three of these cases, hypertension reversed with antibiotic therapy [7–10].

The underlying pathophysiological mechanism of pulmonary hypertension in Whipple's disease has not been established but may be directly related to Whipple's disease, with the strongest evidence for a causal relationship being resolution with antibiotic therapy [9]. 

It has been postulated that the accumulation of intraluminal macrophages in the pulmonary arteries and arterioles may lead to a subsequent increase in pulmonary vascular resistance [9]. 

Moreover, it was suggested that adequate control of the inflammatory response can be accompanied by a marked improvement in haemodynamics in certain types of PH [10].

Although Whipple's disease is not generally considered as a possible cause of pulmonary hypertension, such awareness is important because it may be potentially resolved with antibiotic therapy. 
